# Multiomic Characterization of a Rare Case of Pediatric Acute Leukemia With a Novel *ANGPT1::HOXA10-AS* Fusion

**DOI:** 10.1200/PO-26-00409

**Published:** 2026-09-01

**Authors:** Haley Newman, Derek Wong, Jeffrey Schubert, Jinhua Wu, Juan Santana, Ariel Long, Zirui Zhou, Feng Xu, Jiani Chen, Moe Takeda, Lisa Eidenschink Brodersen, Kathrin M. Bernt, Minjie Luo, Sarah K. Tasian, Marilyn M. Li, Yiming Zhong

**Affiliations:** ^1^ Division of Oncology and Center for Childhood Cancer Research, Children's Hospital of Philadelphia, Philadelphia, PA; ^2^ Department of Pediatrics, University of Pennsylvania Perelman School of Medicine, Philadelphia, PA; ^3^ Department of Pathology and Laboratory Medicine, Children's Hospital of Philadelphia, Philadelphia, PA; ^4^ Department of Pathology and Laboratory Medicine, University of Pennsylvania Perelman School of Medicine, Philadelphia, PA

## Introduction

Recent advances in genomic testing have led to refinement of genomic-based risk stratification and designation of low- and high-risk patients with pediatric AML.^1^ However, not all genomic alterations with diagnostic, prognostic, or therapeutic implications can be identified on routine molecular testing.

Long noncoding RNAs (lncRNAs) represent transcripts of over 200 base pairs that cannot be translated into functional proteins because of the absence of open reading frames and have recently been identified as important contributors to AML pathogenesis.^2^ However, lncRNAs are often poorly characterized on clinical DNA-based next-generation sequencing (NGS) and targeted RNA-based fusion panel analyses. Although untranslated, lncRNAs can affect genomic expression via four primary mechanisms via signaling, decoy, guide, or scaffold.^3^ Signal lncRNAs represent developmental stages; decoys bind regulatory proteins and thus repress transcription/translation; guide lncRNAs direct regulatory protein complexes/chromatin modifiers/transcription factors to target sites; and scaffold lncRNAs serve as a location for assemblage and interaction of protein complexes. Herein, we describe a young child with acute leukemia and her clinical case of a previously undescribed AML-associated oncogenic fusion involving lncRNA that was not identified via our standard institutional molecular testing and required whole-transcriptome sequencing (RNA-seq) for identification.

## Patient Case

**FIG 1.**
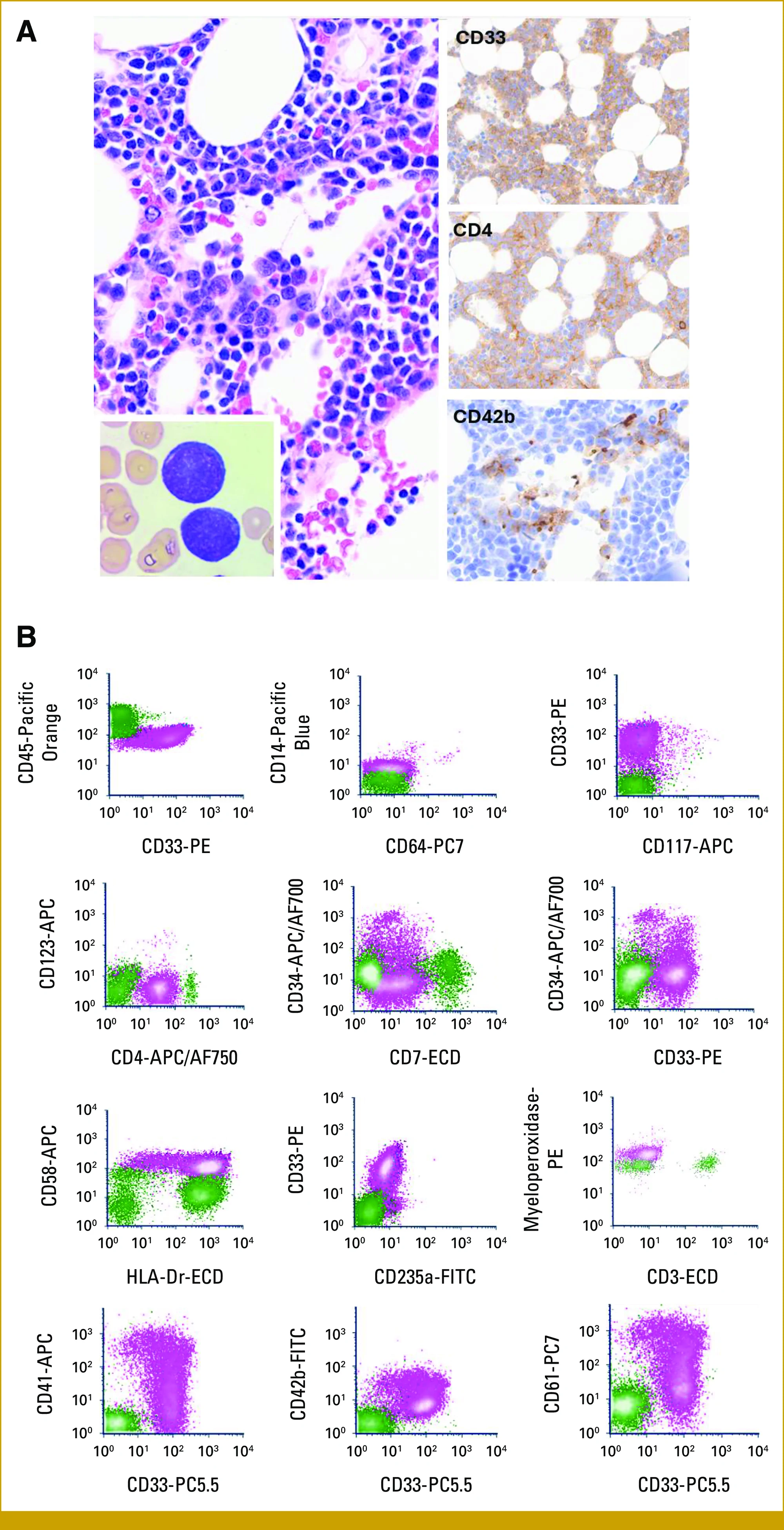
(A) Bone marrow biopsy and aspirate smear. Bone marrow trephine core demonstrated sheets of medium to large, atypical cells, accounting for approximately 50% of cellularity. The neoplastic cells were positive for CD33 and CD4. CD42b highlighted a minor subset of blasts found in small clusters, with a membranous and perinuclear dot-like Golgi pattern. The cells were negative for GATA1, MPO, and CD117 (not shown). Bone marrow aspirate (box insert) smears demonstrated variably sized blasts with round to ovoid nuclei, fine dispersed chromatin, occasional 1-3 small nucleoli, and scant to moderate amounts of azurophilic cytoplasm. (B) Flow cytometry data from the bone marrow aspirate. Abnormal progenitor cells are shown in pink, and mature lymphocytes in green (as an internal normal reference). These abnormal progenitor cells express CD33, CD4, CD58, heterogeneous HLA-DR, and heterogeneous CD34. All other lymphoid and myeloid cell surface antigens were negative (below 20% expression). Heterogeneous expression of CD41, CD42b, and CD61 was observed. CD235a was negative as was myeloperoxidase, along with other cytoplasmic antigens tested.

A 13-month-old previously healthy female was referred to hematology for isolated thrombocytopenia noted on routine CBC screening at her 1-year well-child visit. At the time, she was noted to have experienced progressive weight loss over a period of 2 months, falling from the 63rd to 17th percentile. She was closely monitored by hematology with serial CBCs over the next 6 months, demonstrating persistent isolated thrombocytopenia. Her follow-up CBC at age 19 months was subsequently notable for WBC 16,600/uL, hemoglobin 8.6 g/dL, platelets 13,000/uL, and the presence of 17.4% blasts. Assessments of bone marrow aspirate smears and biopsy were performed (Fig 1A), demonstrating sheets of blasts with irregular nuclei, speckled chromatin, indistinct nucleoli, and scant cytoplasm, accounting for approximately 50% of cellularity. The aspirate smears demonstrated variably sized blasts with round to ovoid nuclei, fine dispersed chromatin, occasional 1-3 small nucleoli, and scant to moderate amounts of azurophilic cytoplasm, whereas the bone marrow trephine cores demonstrated sheets of medium-sized to large blasts with irregular to ovoid nuclei, speckled chromatin, indistinct nucleoli, and scant cytoplasm, accounting for approximately 50% of cellularity. Flow cytometric analyses of the peripheral blood and bone marrow aspirate demonstrated a blast population positive for CD58, CD33, CD4(dim), CD71, and HLA-DR. The blasts lacked expression of cCD3, CD7, CD19, CD13, CD117, MPO, CD15, and other B-cell and T-cell markers. Immunohistochemical staining of the bone marrow biopsy demonstrated CD33 and CD4 positivity of leukemia blasts, whereas GATA1, MPO, and CD117 were negative. CD42b highlighted a minor subset of blasts, found in small clusters, with a membranous and perinuclear dot-like Golgi pattern. Reticulin stain demonstrated grade 1 fibrosis. While the expression of CD41, CD42b, and CD61 was negative on the majority of cells, approximately 40% of cells demonstrated heterogeneous expression of these megakaryoblast-related markers (Fig 1B). Interpretation of expression of these markers is technically challenging as they often exhibit nonspecific staining on maturing monocytes and myeloid cells. Overall, the immunophenotype was suggestive of an acute myeloid lineage leukemia, but precluded definitive classification, leading to a pathologic diagnosis of acute undifferentiated leukemia.

**FIG 2.**
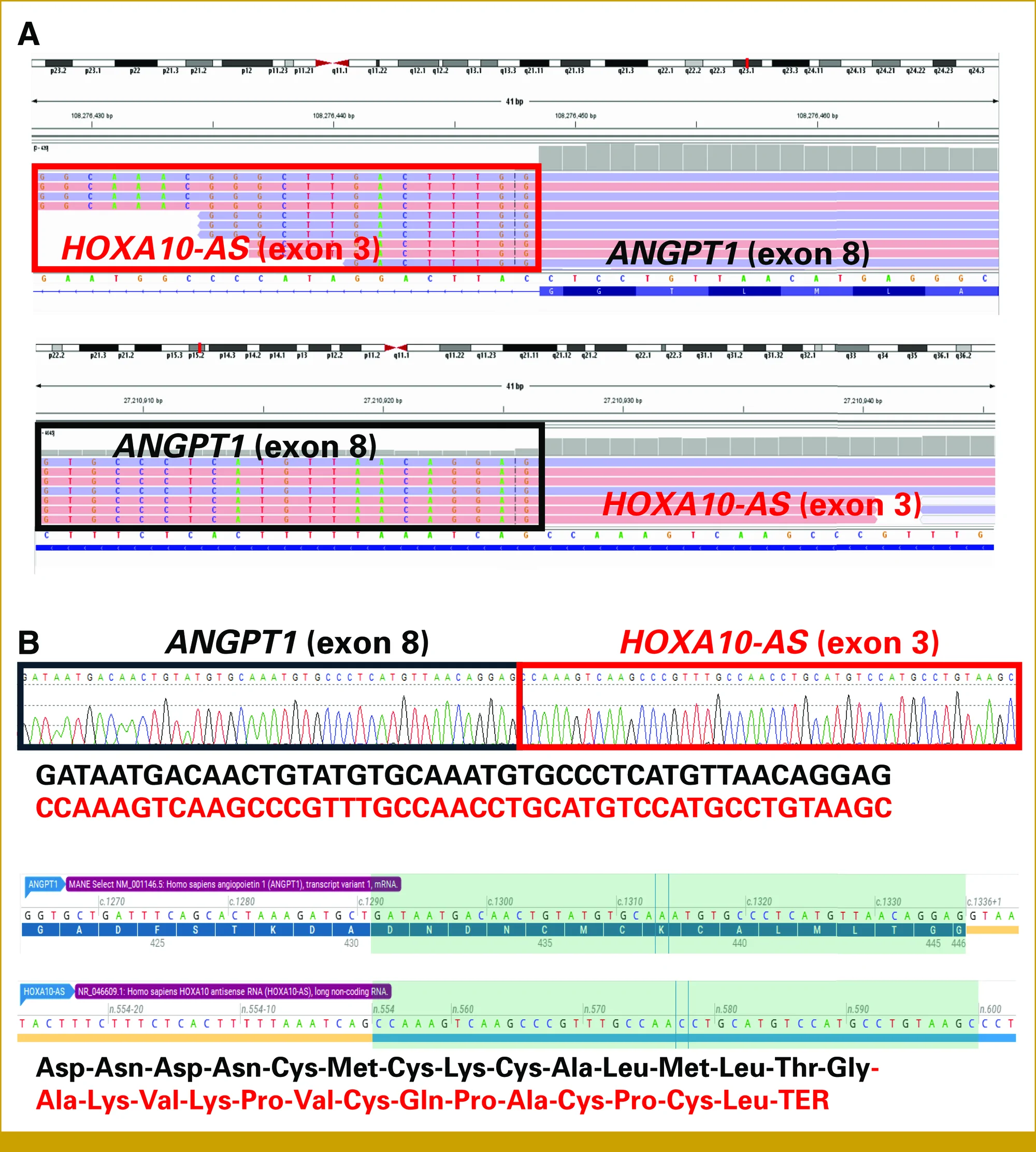
(A) Integrative Genomics Viewer (IGV) screenshots of each breakpoint and the mismatched bases (red and black boxes) which highlight the fused sequences. (B) Sanger sequencing of the fusion gene with the black box and text representing *ANGPT1* DNA and protein sequence, and *HOXA10*-*AS* DNA and protein sequence is given in red. Alamut screenshots highlight the location of the breakpoints (*ANGPT1* NM_001146.5 exon 8 on 8q23.1and *HOXA10*-AS NR_046609.1 exon 3 on 7p15.2).

**FIG 3.**
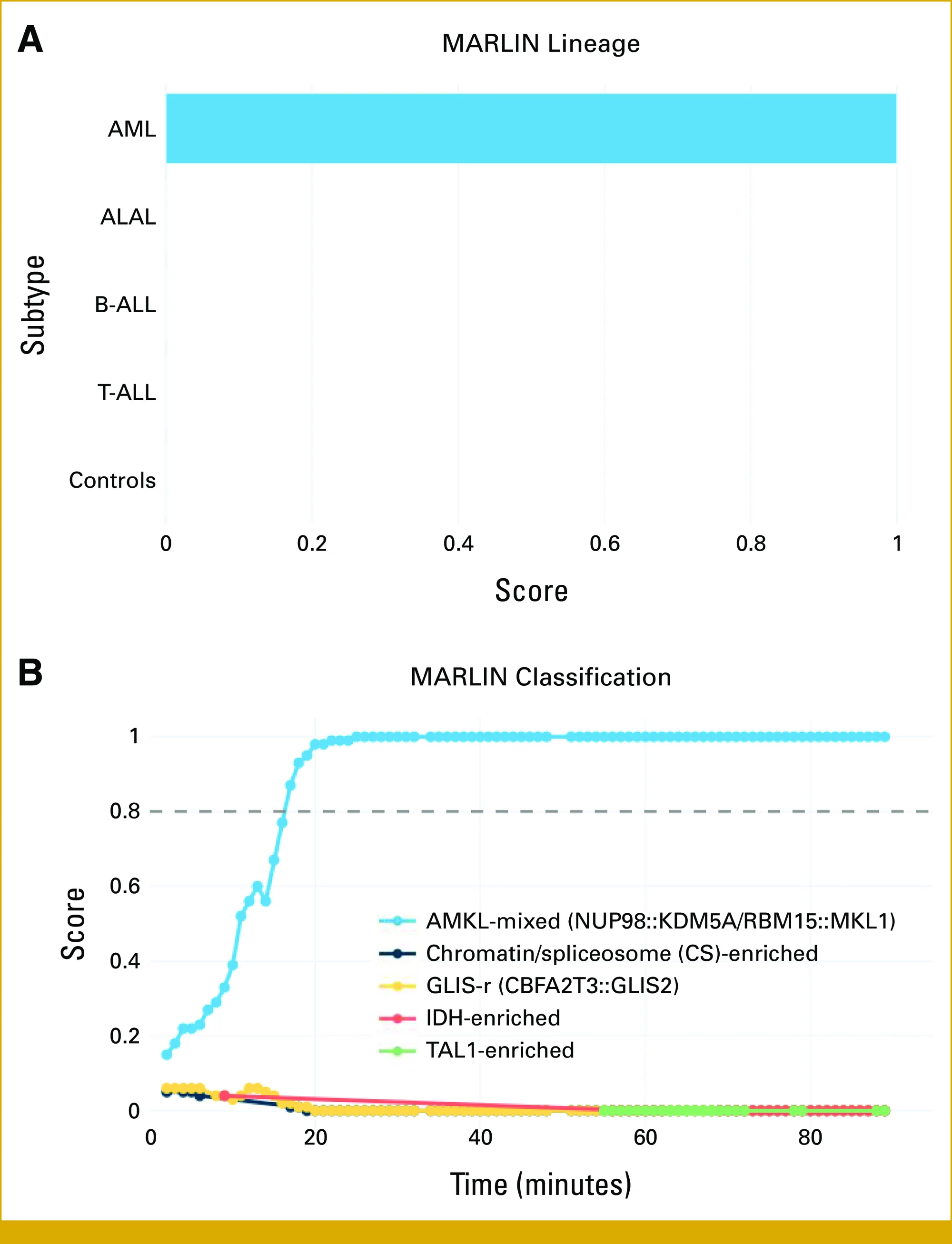
(A) Bar plot showing the lineage prediction by MARLIN. The X-axis represents probability score with 1 = 100% probability. (B) Dot and line plot showing the top 5 leukemia subtype predictions by MARLIN at each minute of sequencing. The dotted line at 0.8 denotes the score at which a classification is deemed confident. The x-axis represents minutes of MARLIN algorithmic sequencing.

Fluorescence in situ hybridization (FISH) analysis using *PML/RARA* and *TP53/RARA* probe sets detected gain of *RARA* (one to two additional copies) and normal signal patterns for *PML* and *TP53* in approximately 65% of cells examined with no evidence of a *PML*::*RARA* rearrangement. Metaphase FISH also demonstrated extra signals for *RARA* on an apparent ring chromosome 17. All other probes, including *RUNX1*/*RUNX1T1*, *KMT2A*, *NUP98*, *MYH11*/*CBFB*, and CEP7/7q31, were negative for copy number alterations or rearrangements. On chromosome analysis, 10 of 17 metaphases contained trisomy of chromosome 2, a complex 4-way translocation involving chromosomes X, 8, both copies of chromosome 7, and a ring chromosome 17 with one intact copy of *TP53* and three intact copies of *RARA*. DNA-based paired tumor/normal NGS panel analysis of 118 genes was performed on bone marrow and skin biopsy (germline) tissues to assess for sequence and copy number variants, and RNA-based fusion panel analysis of 117 genes was also performed on the bone marrow (somatic testing only).^4,5^ Somatic *KRAS* (NM_033360.4: c.35G>C, p.Gly12Ala, VAF = 9%) and *CTCF* (NM_006565.4: c.420_432dup, p.Glu145*, VAF = 10%) sequence variants and copy number alterations consistent with the karyotype (gain of whole chromosome 2, loss of partial 17p, complex gains and losses on 17q) were detected via this testing. However, no clear leukemia molecular driver was identified. Whole-transcriptome sequencing was thus performed and subsequently identified an *ANGPT1*::*HOXA10-*AS fusion (Fig 2A), which was confirmed by Sanger sequencing (Fig 2B). Increased gene expression of HOX family members and transcriptional cofactors as compared with the bone marrow control cohort, including *HOXA10*, *HOXA-AS*, *HOXB-AS1*, *MEIS1*, and *MEIS2*, was also identified (Appendix Table A1). Nanopore long-read sequencing was performed on a research basis for further characterization of the leukemia. The MARLIN classifier, a machine learning–based algorithm to determine acute leukemia classification based on methylation signatures, was implemented based on the nanopore results. For this sample, MARLIN predicted AML lineage with subtype classification as acute megakaryoblastic leukemia (AMKL; Fig 3).^6^ This specific methylation class (AMKL-mixed) encompasses leukemias that exhibit megakaryoblastic differentiation and are driven by various alterations, but excludes the *CBF2AT3*::*GLIS2* fusion prevalent in young children with AMKL and associated with chemoresistance and poor long-term survival.^7^

The child initiated induction 1 chemotherapy with daunorubicin, cytarabine, and gemtuzumab as per the Children's Oncology Group AALL1831/arm A clinical trial regimen and achieved flow cytometric measurable residual disease (MRD)–negative remission (<0.02%).^8^ Given her favorable induction response, hematopoietic stem-cell transplantation in first complete remission was not indicated and the patient was planned to receive a total of five chemotherapy cycles.^9^ Because of severe aspergillus pneumonia infection in induction 1, she received a cycle of less-myelosuppressive azacytidine and venetoclax before resuming cytarabine and anthracycline–based induction and intensification chemotherapy on improvement of infectious complications.

## Ethic Statement

This study was approved by the Institutional Review Board of the Children's Hospital of Philadelphia (CHOP; IRB#17-014759). The requirement for written informed consent was waived by the IRB.

## Discussion

*ANGPT1::HOXA10-AS* is a fusion involving the 5′ region of *ANGPT1* and the lncRNA *HOXA10-AS4*. To our knowledge, the *ANGPT1*::*HOXA10-AS* fusion detected in our patient is novel and is predicted to activate intracellular HOX/MEIS programs. Fusion genes involving the HOX gene cluster (often with upregulation of adjacent HOX family genes) have been reported in approximately 15% of cases of non–Down syndrome-related AMKL (ML-DS).^10^ In one report, most fusions were predicted to lead to an in-frame functional protein. Other fusions involve noncoding HOX antisense genes and may promote leukemogenesis by disrupting regulatory transcripts leading to increased expression of neighboring *HOXA* genes.^11^ *ANGPT1* is normally expressed in endothelial cells and has strong promoter/enhancer activity. *HOXA-AS4* is a lncRNA which functions in *cis* regulation of *HOXA* expression. This fusion likely results in deregulated expression of HOXA cluster regulatory elements. Although this fusion was not covered by our institutional fusion panel, it was identified along with increased *HOXA* expression via whole-transcriptome sequencing. In one review examining drivers of non–Down syndrome-associated AMKL, diverse rearrangements in the *HOX* loci sharing gene expression profiles had relatively favorable prognosis as compared with other subgroups (60-month overall survival 77%), although limited by small numbers.^10^

Knowledge of this alteration might have therapeutic implications. Aberrant expression of HOX family genes and *MEIS1* leads to arrested differentiation and interaction between KMT2A and menin. Menin inhibitors block this interaction and have demonstrated clinical efficacy in patients with *KMT2A*-rearranged, *NUP98*-rearranged, *MN1*-rearranged,^12^ *NPM1*-mutant, or *UBTF* tandem duplication.^13,14^ Addition of the menin inhibitors revumenib or ziftomenib to chemotherapy was considered for this child given her leukemia-associated genetic alterations, but was ultimately not pursued given her favorable response to standard-of-care induction chemotherapy. Menin inhibitor–based salvage therapy certainly would be considered were the child to experience leukemia relapse, with the goal of achieving deep MRD-negative pretransplant remission. Additional nanopore sequencing of our patient's case provided methylation classification of her leukemia as AML with AMKL features, consistent with RNA-seq genomic findings. Although nanopore sequencing is currently only available on a research basis, such testing may be helpful for future clinical use in cases in which pathology and immunohistochemistry are nondefinitive for leukemia subtype classification.

In summary, this case illustrates the utility of whole-transcriptome sequencing for identification of genomic drivers and prognostic and therapeutic potential for future implementation of a methylation-based classification for rapid identification of leukemia subtypes.

## APPENDIX

**TABLE A1.** Gene Expression Changes Compared With the Bone Marrow Control Cohort

Gene	TPM	Fold Change	Log_2_ Fold Change
*MEIS2*	20.07	238.76	7.90
*MEIS1*	80.47	59.44	5.89
*HOXB-AS1*	1.25	71.75	6.16
*HOXA10*	50.46	73.67	6.20
*HOXA-AS4*	6.54	108.65	6.76

Abbreviation: TPM, transcripts per million.
